# Lysosome: not only for digestion but also for production

**DOI:** 10.1093/lifemeta/loag007

**Published:** 2026-03-21

**Authors:** Life Metabolism Editorial Team

**Figure 1:**
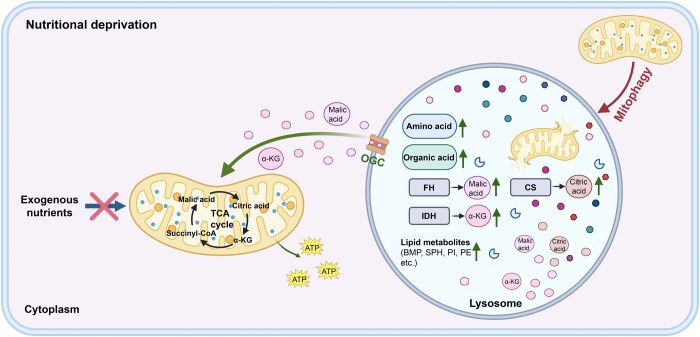
Schematic model of lysosomal metabolic reprogramming and the maintenance of TCA-related metabolites under nutrient deprivation. During energy stress, hippocampal lysosomes may act as “substrate sinks” by accumulating amino acids, specific lipids (e.g. bis(monoacylglycero)phosphate [BMP] and sphingosine [SPH]), and TCA-related metabolites. Based on subcellular localization, this lysosomal TCA metabolite pool is modeled by three parallel pathways. (ⅰ) Mitophagy-mediated delivery: autophagic engulfment delivers mitochondrial components (e.g. citrate synthase and citric acid) into the lysosomal lumen. (ⅱ) Local generation: signals consistent with lysosome-associated metabolic enzymes (FH and IDH) are compatible with a potential basis for the local production of malic acid and α-ketoglutarate (α-KG). (ⅲ) Transporter-mediated exchange: signals consistent with the oxoglutarate carrier in LAMP1^+^ compartments suggest a possible route for cross-membrane metabolite exchange. Together, these routes outline a working model for lysosome−mitochondria crosstalk during neuronal energy stress. Created in BioRender.

Lysosomes have long been regarded as cellular “degradation centers,” primarily responsible for the breakdown and recycling of macromolecules [1]. A recent study published in *Life Metabolism* suggests that lysosomes may also play a more active role in cellular metabolic organization [2]. By integrating *in vivo* adeno-associated virus (AAV)-mediated lysosomal immunoprecipitation (Lyso-IP) with high-throughput metabolomics [3], this study systemati­cally profiled the lysosomal metabolite landscape of mouse hippocampal neurons under energy stress. The results show that remodeled lysosomes not only accumulate small-molecule substrates and lipids but are also enriched in tricarboxylic acid (TCA)-related metabolites. Coupled with features of mitophagy, lysosome-associated metabolic enzymes, and candidate transmembrane transporters observed within the lysosomal compartment, the study proposes a conceptual model: a “lysosomal TCA metabolite pool” may be collectively maintained by three parallel pathways: mitochondrial delivery, potential *in situ* generation by resident enzymes, and transporter-mediated transmembrane exchange (Fig. 1). Together, these findings suggest that lysosomes may function not only as degradative endpoints but also as adaptive metabolic nodes in the energy-stressed brain.

The brain is a highly energy-demanding organ, and the hippocampus is particularly sensitive to fluctuations in nutrient supply because of its sustained synaptic activity [4]. However, at the *in vivo* subcellular level, how the lysosomal metabolic profile responds to fluctuations in energy status has long lacked direct empirical support. To bridge this gap, the research team leveraged the AAV-LysoTag/Lyso-IP system in conjunction with multi-omics technologies in the mouse hippocampus to delineate the landscape of lysosomal metabolism and lipid remodeling under energy stress. This high-resolution subcellular atlas not only supports a role for lysosomes as substrate reservoirs and sites of lipid remodeling during energy deprivation but also offers new clues to their association with TCA-related metabolism.

This work is notable in three respects. First, it expands the ­traditional understanding of lysosomes as degradation endpoints by proposing that they can also serve as a “substrate sink” during energy shortage, thereby elevating their role to an adaptive, potential “metabolic hub.” Second, based on the multi-pathway mechanisms of mitochondrial delivery, local catalysis, and transporter exchange, it deepens our view of lysosome−­mitochondria crosstalk in maintaining neuronal metabolic homeostasis. Finally, the direct biochemical profiling of lysosomal contents provides a prospective strategy for the classification of lysosomal subpopulations based on “metabolic states.” Traditionally, the classification of the endosome−lysosome system relies heavily on static surface membrane components, such as Rab5/PI3P labeling early endosomes, Rab7 labeling late endosomes, and LAMP1/2 together with lipids such as BMP labeling mature lysosomes [5]. However, ­relying solely on outer membrane markers makes it difficult to finely delineate lysosomal subpopulations with specific biochemical functions [6]. This study suggests that future research could move beyond classifications based solely on membrane markers. By leveraging highly sensitive metabolite fluorescent probes developed by the scientific community, combined with LAMP1 co-localization and live-cell imaging technologies, researchers could visually distinguish specific metabolic subpopulations from general degradative subpopulations of lysosomes at the single-cell level [7]. This perspective compensates for the limitations of static surface markers, propelling research on organelle heterogeneity towards the dimension of internal ­biochemical functions.

Although the study suggests that lysosomes exert an active regulatory role within the neuronal metabolic network, several mechanistic questions now come into focus. First, the specific targeting pathways by which metabolic enzymes lacking typi­cal signal peptides, such as fumarase (fumarate hydratase, FH) and isocitrate dehydrogenase-1/2 (IDH1/2), reach lysosomes remain to be verified. For example, it is not yet clear whether they enter via chaperone-mediated autophagy or unconventional secretion. Second, it remains unknown how these enzymes evade degradation and maintain catalytic activity within the acidic, hydrolase-rich lysosomal lumen. Whether this survival relies on specific post-translational modifications or physical sequestration within lipid microdomains warrants further investigation. Third, the sources of upstream substrates required for localized enzymatic reactions and the export pathways for downstream products must be clarified to construct a complete transmembrane material-flow circuit. Fourth, there is a need to use high-resolution live-cell imaging technologies to resolve the spatiotemporal crosstalk among multiple organelles, including the endoplasmic reticulum, mitochondria, and lysosomes, during energy fluctuations. Fifth, the broader applicability of this mechanism awaits validation. Recently, a whole-brain lysosomal proteome atlas published in *Cell* by the Abu-Remaileh group at Stanford University also identified metabolic enzymes within lysosomes of various brain cell types [8]. Together, these observations raise the possibility that related metabolic features of ­lysosomes may extend beyond the hippocampus and merit broader examination across the central nervous system.

In conclusion, by profiling the metabolic and lipid remodeling landscape of hippocampal lysosomes under *in vivo* energy stress, this study expands the role of lysosomes from mere catabolic endpoints to potential dynamic hubs capable of mobilizing TCA-related metabolites and participating in the maintenance of energy homeostasis. As the field of neurometabolism increasingly pivots towards more complex subcellular dimensions and multidimensional crosstalk analyses, such fundamental work elucidating multi-pathway collaborative networks not only provides a conceptual framework for understanding the metabolic resilience of the brain under extreme conditions but may also inform future efforts to understand how endolysosomal dysfunction emerges in neurodegenerative disease.
